# Unveiling the KRAS Relationship between Affinity and Dynamics: A Molecular Simulations Study

**DOI:** 10.1021/acs.jcim.6c01239

**Published:** 2026-07-16

**Authors:** Nadine Grundschober, Viktorija Dujmovic, Chris Oostenbrink, Dražen Petrov, Zuzana Jandova, Andreas Bergner, Edgar Galicia-Andrés

**Affiliations:** † 27270 BOKU University, Institute of Molecular Modeling and Simulation, Department of Natural Sciences and Sustainable Resources, Muthgasse 18, 1190 Vienna, Austria; ‡ 33433 Boehringer Ingelheim Pharma GmbH & Co. KG, Dr. Boehringer Gasse 5-11, 1121 Vienna, Austria; § Boehringer Ingelheim Pharma GmbH & Co. KG, Birkendorfer Str. 65, 88397 Biberach, Germany; ∥ Christian Doppler Laboratory for Molecular Informatics in the Biosciences, BOKU University, Muthgasse 18, 1190 Vienna, Austria

## Abstract

Kirsten rat sarcoma virus protein (KRAS) is one of the most important targets in current drug discovery. Its intrinsic properties, including a high structural flexibility and a shallow polar pocket, make KRAS extremely difficult to target. In fact, for a long time it was considered undruggable. A better understanding of its structure and dynamics will help to advance drug discovery efforts. KRAS is a GTPase, which is highly relevant for the growth, proliferation, and differentiation of cells. Several oncogenic mutations have been identified, suggesting that KRAS could be an excellent target in the fight against cancer. In this work, we use molecular dynamics simulations to highlight the challenges of this protein in computational drug design. We start from a characterization of the structural flexibility and the diversity in observed molecular interactions. Then we quantify the thermodynamic reasons for the increased stability of the G12C mutant in the active, GTP-bound state. For this we use two thermodynamic cycles and find that the alchemical mutation leads to internally consistent results, in agreement with the biological observations. Furthermore, we explore the use of Accelerated Enveloping Distribution Sampling (A-EDS) to efficiently screen a small set of fragments with the strongest binding affinity. The screening mode of A-EDS already allows for the identification of the most favorable candidate molecules. Subsequently, further optimization of the A-EDS parameters facilitates a quantification of the relative binding affinities in terms of free energies.

## Graphical abstract

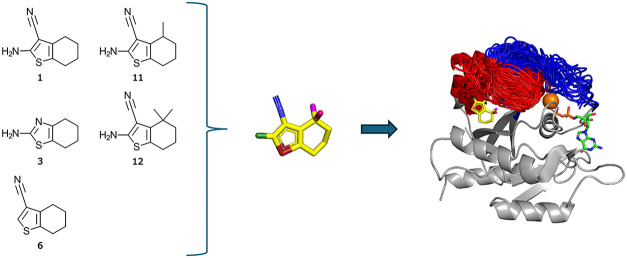

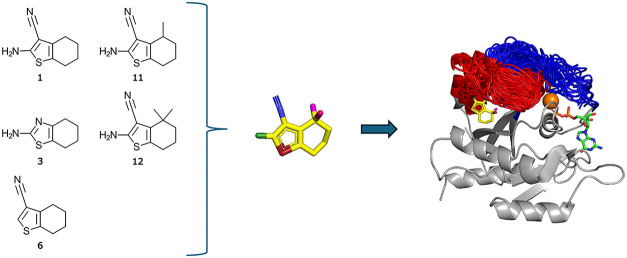

## Introduction

In structure-based drug design, it is crucial to understand the behavior of target proteins because of the direct influence on their specificity, mechanism of action, resistance and mutations.^1–3^ A thorough understanding of the working mechanism of the proteins involved in pathology is crucial to design specific drugs that address issues in e.g., cellular signaling or enzymatic activity.

The Kirsten rat sarcoma virus protein (KRAS), a member of the Ras family of small GTPases, mediates key signaling pathways governing cell growth, proliferation, and differentiation. However, KRAS is prone to suffer from mutations which have been correlated with the development of several types of cancer due to overactivation. The inhibition of KRAS (or more specifically, its oncogenic mutants) has been a challenging task due to the apparent absence of a suitable binding pocket to accommodate effective drugs. KRAS has historically been considered a difficult drug target because of its intrinsic structural and dynamic properties. The relatively smooth surface with shallow binding pockets and its high flexibility as a result of its sII and sI/sII loops make it difficult for small molecules to bind with high affinity and selectivity. Recent advances have produced promising inhibitors, yet much of this progress is still largely limited to the G12C mutant and does not readily extend to many other common oncogenic KRAS variants.^4,5^ For this reason, KRAS was long considered an “undruggable” target.^6–8^

The first KRAS inhibitors emerged in 2012, when the binding of small ligands to a newly identified pocket was discovered.^7,9^ Due to limited cellular efficacy and selectivity, these compounds were succeeded by more promising agents directed at the newly characterized switch II pocket, as detailed by Ostrem et al.^10^ This advance ultimately facilitated the development of covalent clinical candidates targeting the inactive GDP-bound KRAS G12C. Covalent modification of the switch I/II region using an inhibitor in conjunction with the S39C mutant enabled fragment-based screening.^11^ This led to the development of a reversible KRAS G12C inhibitor active *in vivo*.^12^ Moreover, recent developments in drug design have led to other strategies such as the tri-complex inhibitor (TCI) modality. This is the formation of a complex bridge between an intracellular chaperone protein CypA and an active GTP-bound KRAS due to the action of the inhibitor (Daraxonrasib), leading to the steric blockage and consequently inhibiting the downstream RAS signaling and tumor cell proliferation.^13^

Pantsar explored widely the interactions of KRAS variants (wild-type and mutants) to understand the interactions involved in the stabilization of ligands;^4^ Chen et al. probed the mutation-induced impacts on structural flexibility, conformational changes and free-energy landscapes of switch domains by using Gaussian Accelerated Molecular Dynamics;^2^ and in 2023, Zhao et al. constructed a vast data set of KRAS structures from the Protein Data Bank (https://www.rcsb.org)^14^ with 256 ligands to characterize binding pockets and ligand interactions, but only performing MD simulations in the apo-, GDP-bound, and AMG510-bound-KRAS structures.^15^ However, to the best of our knowledge, there is still a gap on addressing the challenges for the evaluation of thermodynamic properties, such as binding free energies, due to the flexibility of the protein and the differences in the behavior between various relevant states. Moreover, KRAS’ flexibility can result in inconsistent free-energy measurements; therefore, the need for internal checks becomes a must in such systems.

In this work, we used molecular dynamics simulations to systematically study several factors commonly attributed of influencing KRAS binding affinity to small molecule inhibitors. We focus on the relationship between the flexibility of KRAS, the effect of the oncogenic G12C mutation on its activity and the calculation of binding affinities for a small series of binding fragments, reported by Bröker et al.^12^ Previous works report that most of the challenges, when describing KRAS, come from two highly dynamic loops in the protein structure, the switch I (sI) and switch II (sII) loops.^4,15,16^ Large conformational changes in these loops facilitate the binding to small molecules, which poses significant challenges for prospective structure-based drug design. To assess the effect of such conformational diversity, we reproduced the typical affinity of KRAS wild-type and G12C mutant for GDP and GTP nucleotides and assessed the impact of flexibility on ligand affinity. Moreover, we used free-energy calculations as a rigorous internal validation, enabling the identification of reliable results in pathways where molecular flexibility significantly impacts binding affinity. Finally, we present a case study that exhibits the robustness of A-EDS to efficiently screen several ligands and to quantify the relative binding free energies.

In drug design, alchemical free-energy methods are widely used to calculate the relative binding free energies of different ligands to a target protein. These methods, such as Free-Energy Perturbation (FEP) and Thermodynamic Integration (TI), rely on the gradual transformation of the Hamiltonian function of one ligand into another, typically by introducing several non-physical intermediate states (λ-states). However, one of the challenges is the efficient description of slow relaxation processes, such as loop re-arrangements, as these processes need to be sampled across all λ-states. Accordingly, extensive simulation times are necessary in each λ-state.

An alternative approach is Accelerated Enveloping Distribution Sampling (A-EDS), where a single reference state is constructed to simultaneously sample all relevant end-states within a single simulation.^17–19^ This is achieved by using a reference Hamiltonian, which combines the Hamiltonians of the end-states, weighted according to their respective Boltzmann factors. When considering the flexible loops of KRAS and their slow relaxation dynamics in MD simulations, calculating the binding affinity of a set of ligands using pairwise free-energy methods requires excessively long simulation times. To address this limitation, A-EDS was applied as a proof of principle in this work, enabling the calculation of the binding affinities for a series of small drug precursor fragments within a single simulation. The binding characteristics of these fragments throughout various stages of optimization are discussed further in the section addressing relative binding free energy in this work.

## Methodology

We performed analyses of the factors influencing KRAS dynamics, focusing on its functional switching mechanism. We consider the bound nucleotides, key activating mutations, as well as interactions of KRAS with small-molecule inhibitor fragments.

The starting point for all simulations was the crystal structure of KRAS with a Cys mutation at the Gly12 position (G12C). The structure was retrieved from the RCSB protein data bank with the PDB code 8AFC, containing the nucleotide Guanosine-5′-diphosphate (GDP) bound to a (Mg^2+^) cation, the ligand 2-amino-4,4-dimethyl-4,5,6,7-tetrahydro-1-benzothiophene-3-carbonitrile (compound **12**) and a phosphate (PO_4_^3–^) anion, which was discarded in our G12C model. The wild-type model (WT) was build from the G12C model by substituting the Cys12 residue for Gly. A similar procedure was followed to generate the Guanosine-5′-triphosphate (GTP) model, by adding manually the γ-phosphate to the GDP model. All models containing the compound **12** at the sII pocket are referred to as Holo structures, whereas without the compound as Apo structures.

Molecular dynamics simulations were carried out using the GROMOS11 ^20^ and GROMACS^21^ simulation packages: GROMACS was chosen for its computational efficiency whereas GROMOS11 for its implementation of the A-EDS method. Using the same parameters and protocols in both MD engines led to consistent results. The molecular interactions of KRAS, nucleotides, ligands, and ions were described using the GROMOS 54A8 force field,^22,23^ whereas the SPC model was used for water molecules.^24^ The protonation state of the amino acids was selected according to a pH value of 7. The structure and dynamics of the systems were analyzed using the gromos++ set of analysis programs.^25^

### Molecular Dynamics Simulations

Table 1 summarizes the different systems that were simulated. For each of these systems, the protein was placed in a cubic box of volume 6.7 × 6.7 × 6.7 nm^3^, solvated in water (∼9200 water molecules) and neutralized in a physiological NaCl solution of 150 mM (*i.e.*, 28 Cl^–^ and 34 Na^+^ counterions for GDP; 35 Na^+^ for GTP).

**Table 1:** Summary of Simulated KRAS Systems^a^

System	Nucleotide	Apo/Holo
G12C	–	(Apo)
G12C	GDP	(Apo)
G12C	GTP	(Apo)
G12C	GDP	Compound **12** (Holo)
G12C	GTP	Compound **12** (Holo)
WT	–	(Apo)
WT	GDP	(Apo)
WT	GTP	(Apo)
WT	GDP	Compound **12** (Holo)
WT	GTP	Compound **12** (Holo)

a Five 1-μs simulations were performed for every system.

All systems were minimized with the steepest-descent algorithm. Subsequently, systems were equilibrated for 1 ns with a time step of 2 fs at constant volume and temperature of 300 K and relaxation time of 0.1 ps using the weak-coupling thermostat.^26^ For the production stage, every system was simulated in five independent replicate simulations of 1 μs each. The temperature and pressure of the systems were kept constant at 300 K and 1 bar using the Velocity-rescale and Parrinello–Rahman algorithms with relaxation times of 1.0 and 2.0 ps, respectively. The simulation box was scaled isotropically with an isothermal compressibility of 4.5 × 10^–5^ nm^3^ mol kJ^–1^. Long-range interactions were handled with the reaction field, a cutoff radius of 1.4 nm and dielectric constant of 61. All bonds were constrained with the SHAKE and LINCS algorithms.^27,28^

The overall flexibility of the simulations was analyzed in terms of the atom-positional root-mean-square fluctuations (RMSF). The binding site pockets were characterized based on the size, shape, accessibility and hydrophobicity using the MDpocket package,^29^ as described by Kuvek et al.^30^ Furthermore, the interactions of the ligand and the nucleotide were quantified in terms of distance heatmaps and the occurrence of hydrogen bonds, as well as the relative binding free energy, as described in the next section. The presence of hydrogen bonds was determined using a geometric criterion, *i.e.*, a hydrogen bond was observed if the hydrogen-acceptor distance was below 0.25 nm and the donor-hydrogen-acceptor angle was at least 135°.

### Free-Energy Calculations

#### Effect of G12C Mutation on the Nucleotide Affinity

The preference of the wild-type KRAS protein and the G12C mutant for the nucleotide GTP vs GDP was assessed in terms of the relative binding free energy, as calculated from the thermodynamic cycle that involved either the alchemical modification of GDP to GTP, or the alchemical mutation of glycine (Gly; G) to cystein (Cys; C) at position 12 of the protein. These calculations were performed using the FEP method by employing a coupling parameter λ, connecting an initial state A to a final state B. We initially used 21 λ-values, each simulated for at least 2 ns. The free-energy difference between states was computed using the Bennett Acceptance Ratio (BAR) method.^31^ To improve the convergence of free energies, we increased the number of λ-values and extended the simulation time (2 ns per every new λ-iteration, with a maximum of four iterations). We performed the GDP to GTP modification for the nucleotide free in solution and when bound to both the wildtype and the G12C mutant. Similarly, the G to C mutation was performed in a protein without nucleotide bound and with either GDP or GTP bound. These six different perturbations were implemented in both forward and backward directions, and every perturbation was performed in three replicas.

#### Relative Binding Free Energy of a Small Set of Ligand Fragments

To furthermore calculate the relative binding free energy of five small variations of the ligand 2-amino-4,4-dimethyl-4,5,6,7-tetrahydro-1-benzothiophene-3-carbonitrile (compound **12**), we used the Accelerated Enveloping Distribution Sampling (A-EDS) method.^18,19^ We applied A-EDS to five fragments reported by Bröker et al., which were used to design the inhibitor *BI-0474*.^12^ The fragments, shown in Figure 1, were selected to test the A-EDS method, explicitly including both the R- and S-configurations of compound **11**.

**Figure 1:**
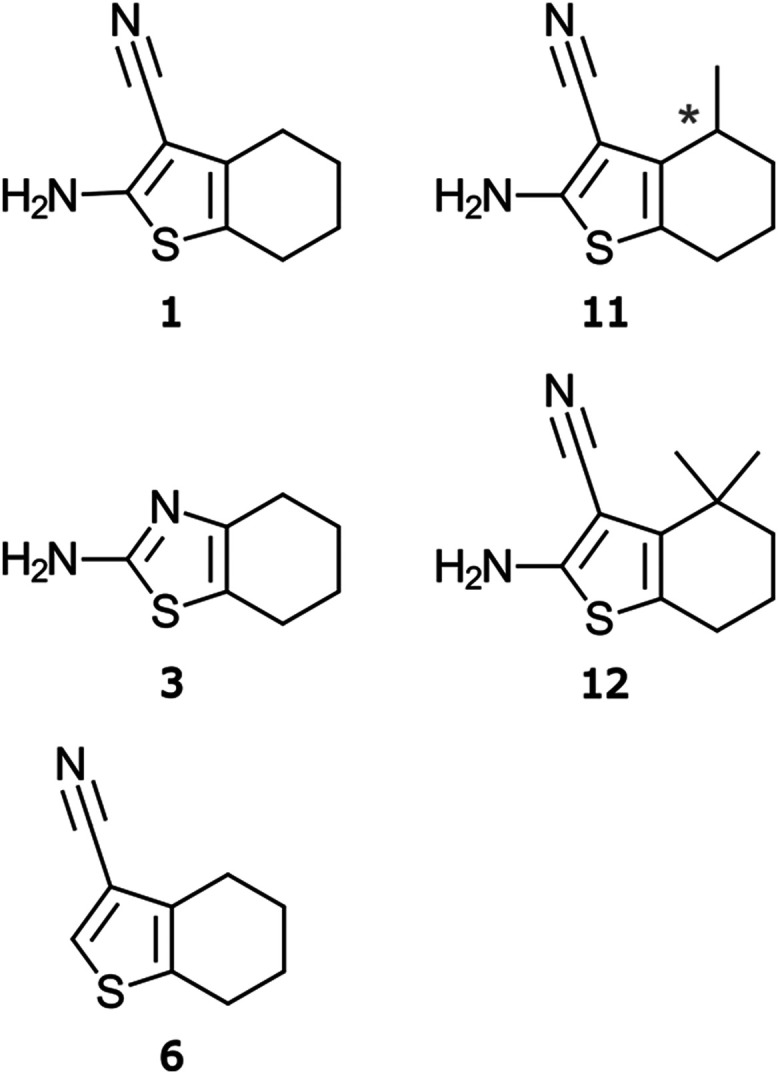
Selected fragments to compute relative affinities to the KRAS G12C mutant. Fragment and compound numbering are according Bröker et al.^12^ Compound **11** was included in both the R- and S-configuration, indicated with an asterisk. Compound **12** corresponds to the inhibitor LXK observed in the X-ray structure with PDB code 8AFC.

The A-EDS method is based on simulating an unphysical, hybrid state that is representative of a series of compounds simultaneously. To generate this hybrid reference state, the molecular topologies of the six fragments in Figure 1 were merged using the python package SMArt.^32^ Any additional atoms required to represent all end states are included as dummy atoms. The reference state, representing the six compounds, was solvated in a rectangular water box with a minimum distance of 1.4 nm from the solute to the box walls. The simulation procedure is the same as the one described in the free-energy calculations section.

The energy of each of the six compounds that makes up the A-EDS reference state is monitored during an A-EDS simulation. The compound with the lowest energy is considered to be sampled at that particular simulation step. The A-EDS method starts with a non-equilibrium parameter search with the aim to ensure the frequent and roughly equal sampling of each of the six compounds. It determines energy offsets for the six states, simultaneously with the acceleration parameters, *E*_min_ and *E*_max_.^19^ In this work, a search simulation of 50 ns was performed for the reference state in water.

The reference state was subsequently placed in the protein binding site, by superposition of the co-crystallized ligand, compound **12**. An additional 50-ns A-EDS search was performed in which the offset parameters of the six compounds were readjusted to ensure equal sampling in the protein environment. In this search, the acceleration parameters were kept to the values obtained from the water simulation.

Two equilibrium simulations of 50 ns each for the reference state in the protein environment were performed, employing: (1) the set of A-EDS parameters determined during the search simulation in solvent; and (2) the updated set of offsets from the search in the protein. If the set of parameters obtained during the search simulation in solvent leads to equal sampling in water, the A-EDS approach offers the possibility to screen for the best binders by using exactly these A-EDS parameters in the protein environment. In the protein, an unequal sampling of the individual compounds, immediately separates good from bad binders, as the more favorable compounds can be expected to be sampled more often. Consequently, the preferred compounds can immediately be determined.^33^ Here, the sampling time of each compound was used to score the affinity of the compounds. In the equilibrium simulation with the updated offset parameters (simulation 2), a more equal sampling of the six compounds is expected, which allows us to calculate the relative binding free energy quantitatively.^19^

## Results

### Flexibility of the Switch Loops

The atom-positional root-mean-square fluctuations (RMSF) of the KRAS backbone for the simulation of the G12C mutant, with GDP and compound **12** bound is shown in Figure 2a. The highest fluctuations are encompassed in the residue regions 30–40 and 58–72, corresponding to the sI and sII loop structures, respectively, according to Pantsar’s definitions.^4^ Figure 2b shows the flexibility of these loops by superpositioning 100 different conformations observed during the simulations. These loops are particularly important, as they determine the formation of the sII pocket, in which compound **12** was observed in the crystal structure.^12^

**Figure 2:**
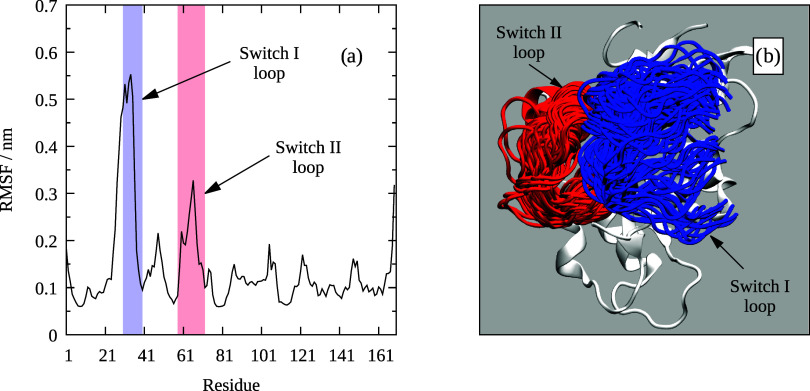
(a) Atom-positional root-mean-square fluctuations of the backbone of KRAS G12C in complex with GDP and compound **12**, with the loop regions indicated. (b) Snapshot of 100 different conformations of sI (blue) and sII (red) loop structures.

Figure 3 compares RMSF profiles from G12C mutant simulations in the presence (Holo) and absence (Apo) of compound **12**. In general, the sII loop region (red) in Apo structures (green line) exhibits greater flexibility than in Holo structures (black line). Although the sI loop region (blue) exhibits higher RMSF values when bound to GDP (Figure 3a) than GTP (Figure 3b), it cannot be concluded that the flexibility is greater due to the fact that the uncertainty estimates overlap with each other. The values in Figure 3 are in good agreement with the RMSF values in the sI loop region reported by Zhao for the KRAS bound to AMG510 and different mutations studied by Mir et al., where the RMSF ranges between 0.3 and 0.4 nm.^15,34^ Similarly, RMSF values observed in the KRAS wild-type oscillate around 0.3 nm with similar ranges (see Figure S1 in the Supporting Information).

**Figure 3:**
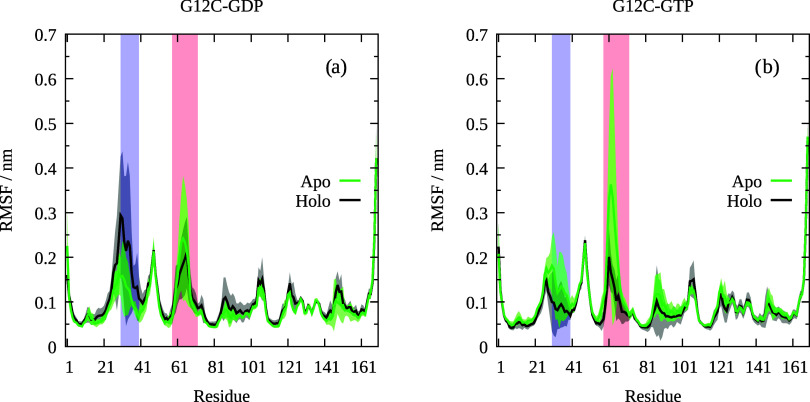
Atom-position root-mean-square fluctuations of KRAS G12C in the Apo (green line) and Holo (black line) states, in the presence of the nucleotides GDP (left panel; a) or GTP (right panel; b). The standard deviation over the five independent replicates is shown as shaded regions. Loop regions sI and sII are highlighted with blue and red colors, respectively.

### Dynamic Pockets in KRAS

Two cryptic pockets have been identified in KRAS, the sII pocket, occupied by compound **12** in the crystal structure (PDB 8AFC) and the sI/sII region as an additional candidate for drug binding.^12^ To analyze the pockets, the trajectory files were filtered by removing the small fragment from the Holo structures, leading to Apo-like structures. Subsequently, all trajectories were analyzed using the MDpocket package.^29^

Due to the flexibility of the sII loop, the sII pocket closes in the Apo simulations, leading to a small range of observed volumes (0.0–4.0 nm^3^), as shown in Figure 4a (pocket sII; Apo). On the one hand, in the presence of compound **12**, its binding site remains more extensive with a volume between 0.2 and 1.0 nm^3^ (pocket sII; Holo). Figure 4b,c display how the increased flexibility of the sII loop significantly reduces the volume of the pocket in Apo simulations. On the other hand, the volume from the crystal structure of the G12C mutant (PDB code 8TY8) in the Apo structure is greater than the corresponding volume range observed dynamically, which emphasizes the importance of dynamic movements of domains, especially those in loops that open and close.

**Figure 4:**
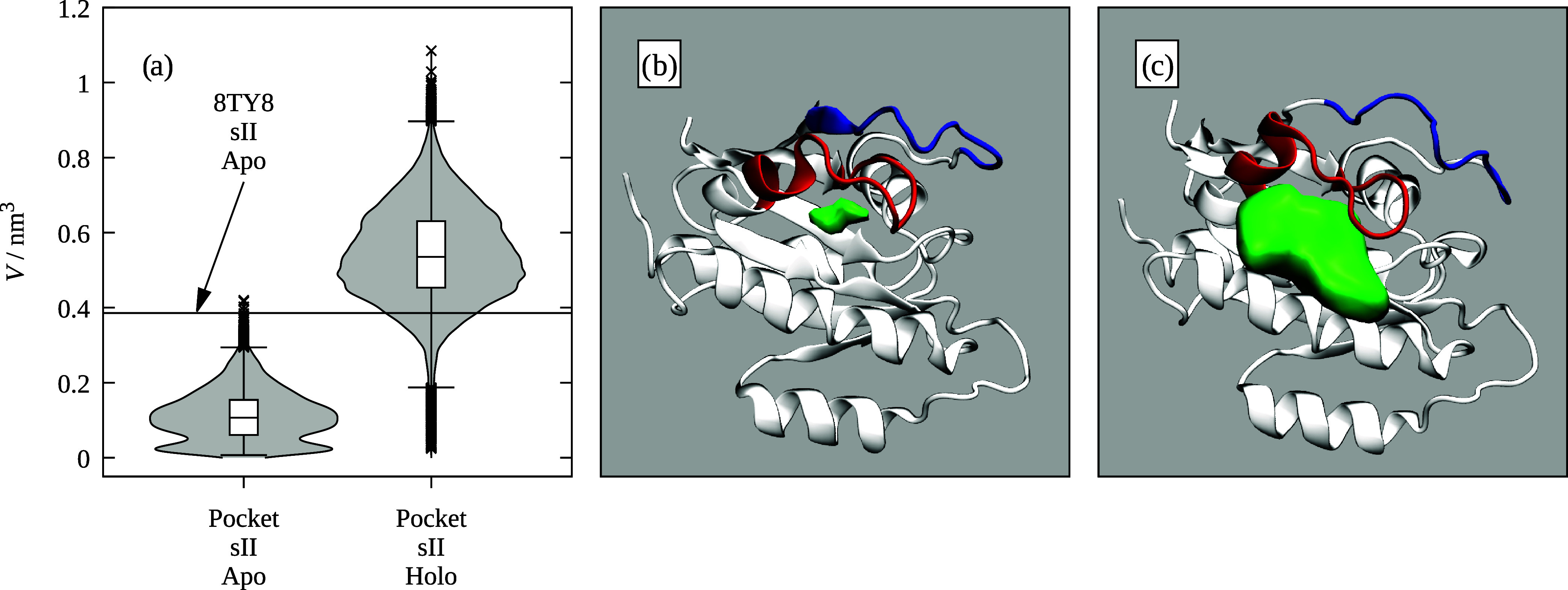
(a) Volume distribution of the pockets sII Apo, sII Holo and sI/II Holo for the G12C KRAS mutant with GDP as bound nucleotide. Representative structures found by MDpocket from the: (b) Apo and (c) Holo simulations. The pockets in (b) and (c) are represented as isosurface volumes with an occurrence frequency in the simulation of 20%. The sI and sII loops are highlighted in blue and red, respectively, as a reference for the pocket. The continuous surface shows the volume size of the crystal structure with the PDB code 8TY8 (G12C mutant; Apo) in the sII loop.

From the output of MDpocket, we determined a range of properties of the pockets, similar to Kuvek et al.^30^ (see Table 2). The shape factor corresponds to the ratio of the area by the volume, normalized to a perfect sphere. A shape factor of 1.0 reflects a spherical pocket, whereas different values correspond to non-spherical pockets. The Apo simulations consistently show low values of the volume and a less spherical active site shape for the sII pocket. Furthermore, these simulations show a higher accessibility, *i.e.*, a higher percentage of conformations in which a connection between the pocket and the external surface of the protein is observed. This is in line with the higher flexibility of the sII loop in these simulations (Figure 3).

**Table 2:** Properties, of the sII Pocket (Top) and sI/sII Region (Bottom) as Observed for the G12C Mutant Following the Definitions in Kuvek et al^30^ ^a^

Nucleotide	GDP	GTP	GDP	GTP
Ligand	–	–	**12**	**12**
sII pocket				
Volume [nm^3^]	0.112 ± 0.068	0.119 ± 0.041	0.541 ± 0.129	0.464 ± 0.130
Volume range [nm^3^]	0.413	0.287	1.060	1.018
Shape factor	0.32 ± 0.21	0.41 ± 0.26	0.96 ± 0.10	0.91 ± 0.13
Accessibility [%]	58 ± 9	54 ± 4	21 ± 10	31 ± 15
Hydrophobicity	0.68 ± 0.25	0.47 ± 0.25	0.59 ± 0.12	0.54 ± 0.16
sI/sII region				
Volume [nm^3^]	0.790 ± 0.416	1.095 ± 0.513	0.804 ± 0.401	0.918 ± 0.437
Volume range [nm^3^]	2.393	3.044	2.315	2.740
Shape factor	0.61 ± 0.32	0.60 ± 0.30	0.63 ± 0.33	0.56 ± 0.28
Accessibility [%]	72 ± 2	65 ± 3	73 ± 7	66 ± 4
Hydrophobicity	0.22 ± 0.24	0.25 ± 0.21	0.19 ± 0.21	0.11 ± 0.17

a The volume describes the average volume of the binding site, the volume range presents the difference between the maximal and minimal volumes. The shape factor represent the normalized fraction of the pocket area by volume. The accessibility displays the percentage of the time that the pocket is accessible from bulk solvent. Hydrophobicity is the fraction of hydrophobic residues lining the pocket.

In contrast, the large volume shown in Table 2 for the sI/sII region, ranging between 2.0 and 3.0 nm^3^, reflects the difficulty of delineating the pocket binding site. This can be seen as the algorithm, in many snapshots, detects the whole protein surface as a binding site, rather than an expected localized volume around residue Val39 (Figure S6). Therefore, the sI/sII region consistently exhibits greater solvent accessibility and lower hydrophobicity values, in line with a shallow, widely open pocket. This is also consistent with a reduced shape factor compared to a sphere-like shape observed for the sII pocket in Holo simulations.

### Molecular Interactions

Molecular interactions were initially assessed in terms of the average value of the shortest atomic distance between two groups of atoms. Figure 5 shows the distance heatmaps of the nucleotides GDP or GTP with amino acids in the residue range 6 – 39, encompassing the residues 10 – 14, defined as the P-loop by Pantsar,^4^ which contains the G12C mutation, and the sI loop. Overall, the simulations with GTP bound show more consistent interaction patterns, especially for the interactions with the sI loop. Of particular interest is the mutant residue Cys12, which exhibits an average distance in a range of 0.3 to 0.4 nm with GDP. However, in the presence of GTP, the average distance reduces to a range of 0.1 to 0.2 nm, suggesting more consistent and stronger interactions. This is in line with the occurrence of hydrogen bonds between Cys12 and the nucleotides, as shown in Figure S2. Almost all hydrogen bonds observed occur between the sulfur atom of Cys12 as proton donor and an oxygen atom from the γ-phosphate of GTP as the acceptor. The possibility of Cys12 in the G12C mutant to form stable interactions with GTP provides a molecular explanation for the shift of the equilibrium states of KRAS toward the active GTP-bound form.^12^ Contrarily, in Figure S3 the distance heatmaps of the wild-type showed a weaker interaction between the nucleotides and both the P-loop and the sI loop with a distance of 0.3 to 0.4 nm from Gly12 and fewer interaction points from the sI loop (see Figure S3).

**Figure 5:**
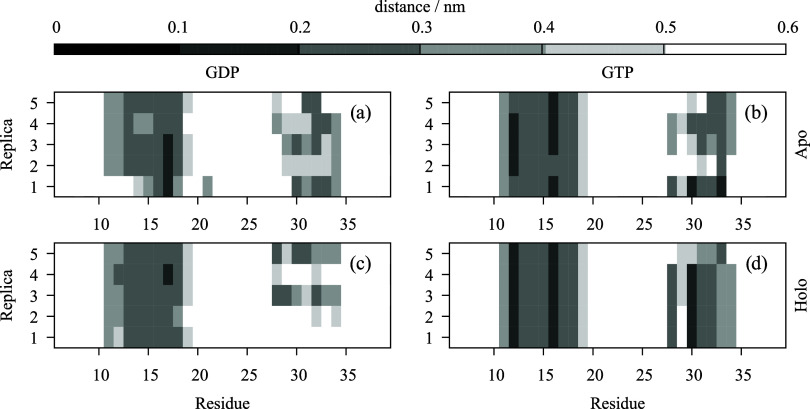
Distance heatmaps between nucleotides GDP (left panels) and GTP (right panels) and residue range 6 – 39 of the G12C mutant Apo (top panels) and Holo (bottom panels) conformations. The average of the shortest distance between the nucleotide and the indicated residues is highlighted for the five individual replicate simulations.

Figure 6 shows the interaction heatmaps for the Mg^2+^ ion, which links the nucleotide to the sI loop and part of the sII loop. Again, more stable interactions are observed in the GTP case, linking the γ-phosphate of GTP to Ser17 and Asp57 of the G12C mutant. We performed additional simulations without Mg^2+^ resulting in an even more strongly fluctuating sII loop, evidencing the key role of the ion plays in preserving the stability of KRAS (data not shown).

**Figure 6:**
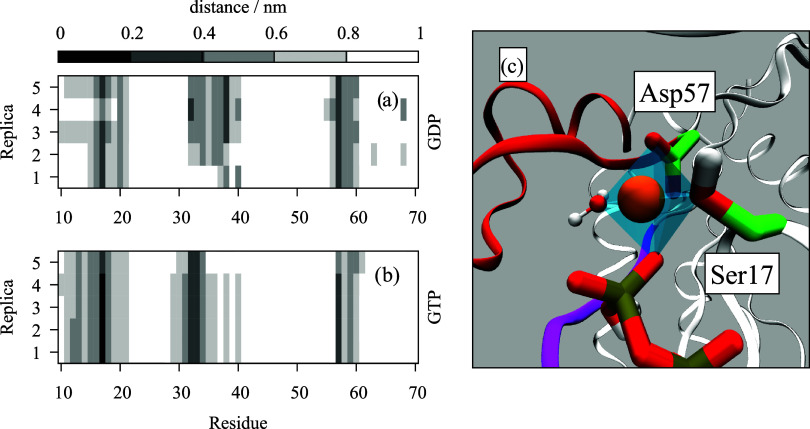
Distance heatmaps between Mg^2+^ and residue range 10 – 70 of the G12C mutant, in complex with compound **12** and GDP (panel a) and GTP (panel b). (c) Representative visualization of the Mg^2+^ coordination in the GTP simulation, with sII loop highlighted in red and P-loop in magenta.

The heatmaps for compound **12** show a diverse behavior in the individual replicates of the simulations (Figure S4). Interactions with the sII loop seem to stabilize the switch, as observed in Figure 3, but the diversity in the replicates suggests that compound **12** does not occupy a unique interacting pose within the pocket. Of all simulations, replica 2 of G12C bound to GDP exhibited the largest number of interactions. The hydrogen bond analysis for individual replicates in Figure 7 shows that the backbone of Glu63 acts as a donor and acceptor for more than 95% of the simulation time in this replica. However, in most of the simulations, alternative hydrogen bonding patterns appear dynamically. Although the same behavior was observed in the wild-type, interestingly more replicas displayed a high occurrence of hydrogen bonds with Glu63 (Figure S5), which is in good agreement with the crystal structure.^12^

**Figure 7:**
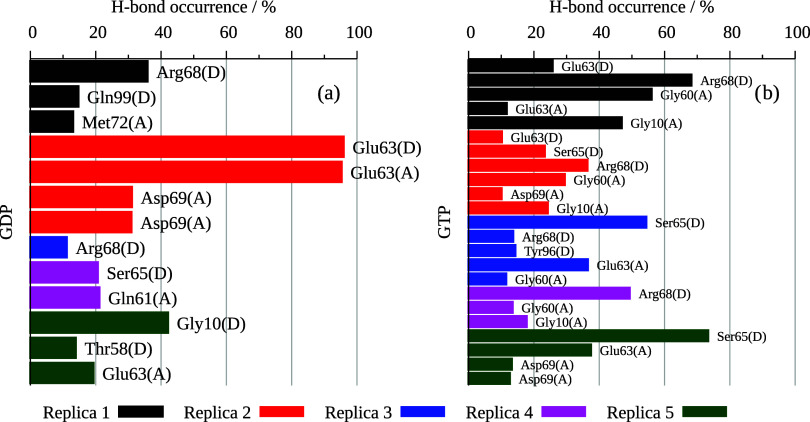
Occurrence of hydrogen bonds between compound **12** and the G12C mutant, in the presence of GDP (left panel) and GTP (right panel). The colors indicate individual replicate simulations.

### Effect of G12C Mutation on the Nucleotide Affinity

To determine the effect of the G12C mutation on the binding affinity of KRAS to the nucleotides GDP and GTP, we performed free-energy perturbation simulations following different alchemical paths as shown in Table 3. On the one hand, we define the forward processes (A → B) of perturbing GDP to GTP as activation and perturbing WT to G12C as mutation. On the other hand, the backward processes are defined as B → A. A visual representation of the thermodynamic cycle is given in Figure 8. We performed the calculations in the forward and backward directions, as this provides a robust check for internal consistency. Since free energy is a state function, the forward and backward processes should yield similar values, differing only in sign. It is clear from Figure 8 that this is not the case for the calculations involving the difference between GDP and GTP when it is bound to the protein. This particular modification involves a charge change, and the simulations did not seem to converge within the four iterations of refinement of the free-energy simulations (see Methodology section). The absence of cycle closure is evidenced by the non-zero sum of the forward and backward Δ*G* values for these modifications in a protein environment reported in Table 3.

**Figure 8:**
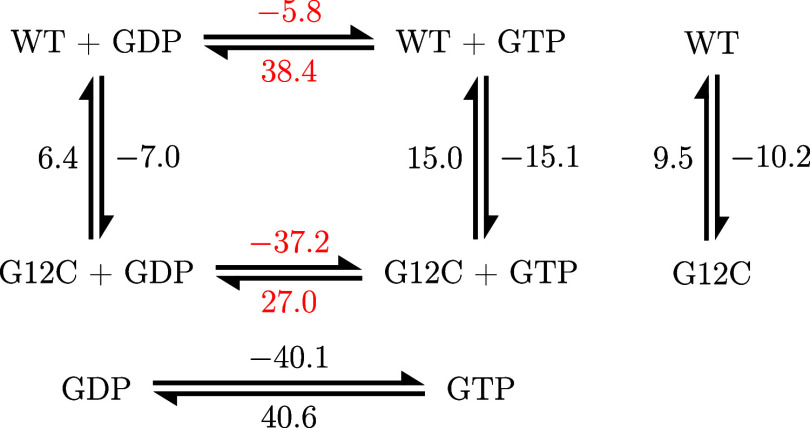
Thermodynamic cycle of KRAS protein in the wild-type (WT) or mutant (G12C) form, bound to the nucleotides GDP or GTP. Numerical values correspond to the free-energy differences (in kJ mol^–1^) from Table 3. The values in red highlight discrepancies observed between the forward and backward calculations.

**Table 3:** Free Energy Differences of Alchemical Modifications Defined by a State A and a State B^a^

State A	State B	Δ*G* ^forward^	Δ*G* ^backward^
(λ = 0)	(λ = 1)	[kJ mol^–1^]	[kJ mol^–1^]
GDP	GTP	–40.1 ± 0.5	40.6 ± 0.5
WT + GDP	WT + GTP	–5.8 ± 0.2	38.4 ± 0.2
G12C + GDP	G12C + GTP	–37.2 ± 0.2	27.0 ± 0.2
WT	G12C	–10.2 ± 0.2	9.5 ± 0.2
WT + GDP	G12C + GDP	–7.0 ± 0.1	6.4 ± 0.0
WT + GTP	G12C + GTP	–15.1 ± 0.1	15.0 ± 0.1

a The free energy difference between the nucleotides GDP and GTP was calculated for the nucleotides free in solution and when bound to the wild-type KRAS (WT) and its G12C mutant (G12C). Similarly, the Gly12 to Cys12 mutation was performed in the absence of any nucleotide, and when bound to GDP and GTP, respectively. The forward perturbation direction is from A to B; whereas the backward direction from B to A. Error estimates are the standard deviations over the three replicate calculations.

By comparing the free energy difference between the wild-type and the mutant (WT → G12C) in the unbound state of the protein and when a nucleotide is bound, the relative binding free energy of the two proteins can be obtained, *e.g.*, for GDP

1 ΔΔGbind(GDP)=ΔGbindG12C−ΔGbindWT=ΔGWT→G12CGDP−ΔGWT→G12C

where the terms in the first line refers to the physically relevant processes and the terms in the second line to the simulated unphysical processes, in the presence and absence of GDP. Using the averages of the forward and backward processes, we find for ΔΔ*G*_bind_(GDP) a value of 3.2 kJ mol^–1^, suggesting that the binding of GDP is 3.2 kJ mol^–1^ less favorable in the G12C mutant than in the wild-type. In contrast, for GTP, we find ΔΔ*G*_bind_(GTP) = −4.9 kJ mol^–1^, suggesting that the mutant binds GTP more favorably than wild-type KRAS. Taken together, the G12C mutation stabilizes the GTP-bound state by −8.1 kJ mol^–1^, offering also a thermodynamic explanation to the observed stabilization of the active state of KRAS, leading to its increased oncogenic behavior. Following the guidelines suggested by Öhlknecht et al. to correct the charge changes,^35^ we performed a *posteriori* free-energy correction on the charge-changing legs of the thermodynamic cycle and found ΔΔ*G*_cor_ between 20.2 and 41.9 kJ mol^–1^. As expected, this does not correct for the discrepancies seen in the hysteresis or cycle closure.

### Relative Binding Free Energy of a Small Set of Ligand Fragments

The free-energy calculations described in the previous section involved at least 21 independent simulations for each process involved. To more efficiently screen the relative binding affinity of a small set of fragments, we explored the use of A-EDS.^18,19,33^ The six structurally similar fragments in Figure 1 (compound **11** in R and S configuration) were combined into a single reference state, which was simulated in aqueous solution and while bound to the G12C mutant of KRAS with GDP also bound.

The simulation of the six compounds in solvent resulted in equal sampling of all the end-states as indicated by the blue bars in Figure 9 and in Table S1. The A-EDS parameters that were optimized in the solvent were subsequently used to probe the sampling time of the fragments in the protein. The green bars in Figure 9 indicate that compounds **12**, **11R** and **11S** are almost exclusively sampled. This very efficiently suggests that these compounds will have the strongest binding affinity within this set of compounds. The screening mode of A-EDS relies on three simulations: (1) search simulation of the A-EDS parameters in water; (2) a production simulation using these parameters in water; (3) a screening simulation using the same solvent-optimized parameters in the protein environment. Using a non-equilibrium search algorithm,^18^ the search simulations in solvent tend to converge readily, facilitating efficient means to screen for the most favorable compounds.^33^

**Figure 9:**
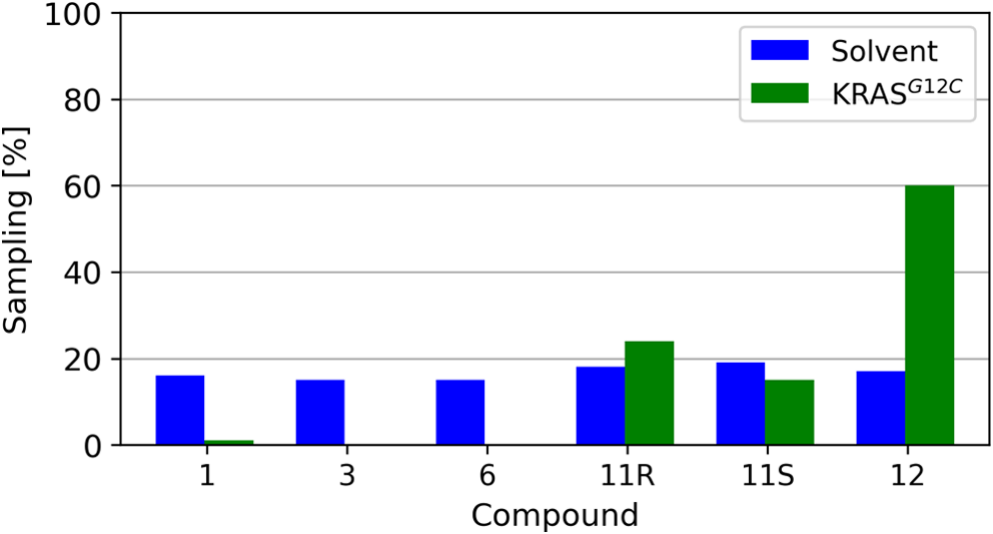
Sampling frequency of the six small fragments from Figure 1 in the production simulation in water (blue) and when using the solvent-optimized A-EDS parameters in the protein environment (green). A shift in the sampling, particularly of compound **12, 11R**, and **11S** in the protein compared to the simulation in water can be observed.

To quantify the binding affinity in terms of the relative binding free energy, an additional A-EDS search simulation was performed in the protein, where the offset parameters for each of the compounds were adjusted to also facilitate the sampling of compounds **1**, **3** and **6**. The relative binding free energy for any compound A, ΔΔ*G*_bind_(*A*), relative to the A-EDS reference state *R*, is computed using

2 ΔΔGbind(A)=ΔGbindA−ΔGbindR=ΔGR→Aprot−ΔGR→Asolv

where the first row defines the binding free energy of compound A (Δ*G*_bind_^A^) relative to the hypothetical binding free energy of the A-EDS reference state (Δ*G*_bind_^R^) and the second row relates this to the free-energy differences between states *A* and *R* in the protein and in solvent. The absolute values in Figure 10 are not directly comparable to any experimental property, but their relative values are. They confirm the data from the screening simulation in Figure 9: compounds **12**, **11R** and **11S** are most favorable, whereas compounds **3**, **6** and **1** show less favorable binding. This agrees well with the trends reported by Bröker et al. for the G12 V mutant of KRAS with a *K*_D_ value for compound **12** below 10 μM while for compound **11** it is 20 μM (without specification of the stereospecificity). For compounds **1**, **3** and **6** the *K*_D_ amounts to 116 μM, 715 μM and >2000 μM, respectively.^12^

**Figure 10:**
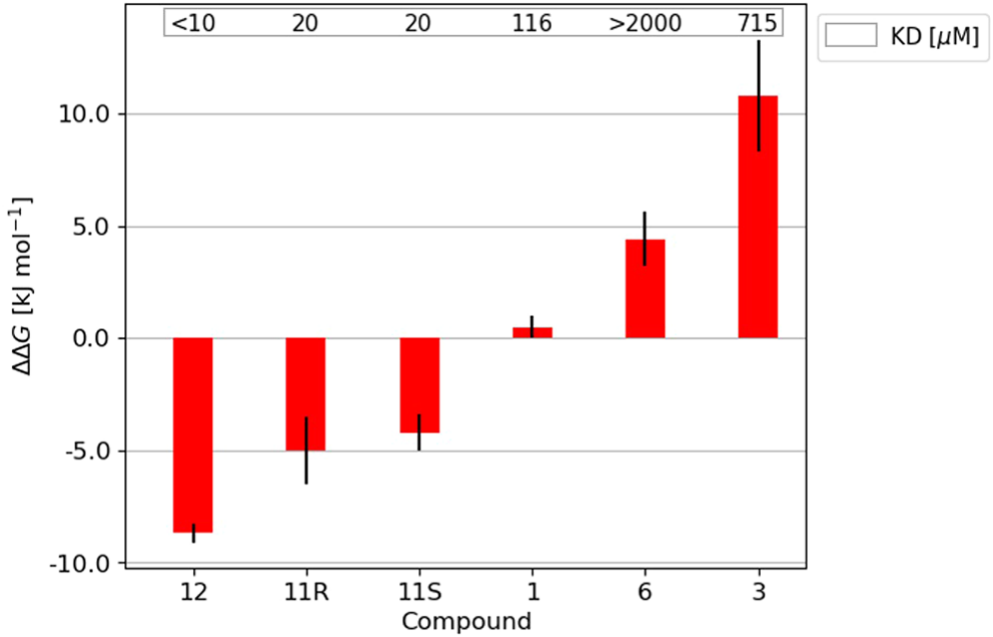
Relative binding free energies, ΔΔ*G*_bind_ for the six fragments of Figure 1. Statistical uncertainties are obtained from block averaging of the simulation data. Negative free energy differences of compounds **11R**, **11S**, **12** show more favorable binding compared to compounds **3**, **6**, and **1**, which follows the same trend as reported in by Bröker et al.^12^ (values in the inset).

## Discussion

Molecular dynamics simulations on various KRAS constructs were performed and analyzed in terms of the structural stability, molecular interactions and thermodynamic properties. The simulations offer molecular and thermodynamic explanations for the effect of the G12C mutation on the activity of the protein. The combination of the G12C mutant, with GTP and compound **12** bound leads to the lowest fluctuations in both the sI and sII loops (Figure 2). This is mainly attributed to the hydrogen bond formed between GTP and Cys12 in the P-loop (Figure S2, as well as varying non-bonded interactions between compound **12** and the sII loop, Figure 7). Additionally, the presence of the compound **12** affects the size and the shape of the sII binding pocket, as the sII loop fills the available space in the Apo structure. Although the sI/sII region also exhibits great potential as a binding site, it remains rather shallow in our simulations, so it appears to be challenging to identify its shape or to design suitable molecules.

Two alchemical approaches were attempted to investigate the relative binding free energies of GDP and GTP in the wild-type and the mutant. The relative binding free energy in the wild-type or mutant protein would be accessible via the alchemical modification of GDP to GTP in the bound and unbound states. However, simulations in the protein-bound states lead to inconsistencies between forward and backward simulations. This highlights the relevance of internal consistency testing. As we performed both forward and backward simulations, we could easily discard these simulations as ill-converged and, therefore, unreliable. Upon analysis of these simulations, we note that the full charge change of the nucleotides requires a relatively large reorganization of the protein structure, *e.g.*, leading to the formation of the hydrogen bond between Cys12 and the γ-phosphate of GTP (Figure S2). This did not occur consistently in the various intermediate simulations that make up the perturbation from GDP to GTP. Note, however, that the perturbation of GDP to GTP and GTP to GDP in water led to consistent results (−40.1 and 40.6 kJ mol^–1^, respectively).

The second approach involves the alchemical mutation of the Gly to Cys. Although simulations involving the glycine residue have previously been seen to be more difficult to converge than, *e.g.*, those involving alanine,^36^ the forward and backward simulations of the current work were in excellent agreement and consistent free-energy differences could be obtained. This allowed us to determine the relative binding free energies of GDP and GTP to wild-type and mutant, and therefore to quantify the stabilization of the GTP-bound state due to the G12C mutation, amounting to −8.1 kJ mol^–1^. Importantly, this result of stabilization of the active, GTP-bound state is in agreement with the oncogenic effects of the G12C mutation.

We furthermore studied how a small fragment bound to the sII pocket affects the stability. The high flexibility of the sI and sII loops (Figure 3), combined with the dynamic behavior of the sII pocket and sI/sII region (Figure 4), accordingly poses challenges for free energy calculations on more than two compounds. In the GDP → GTP modification, we observed that the relevant structural rearrangements need to be observed sufficiently in all the intermediate simulations. Note that the equilibrium simulations of KRAS in Table 1 were performed for five times 1 μs, whereas free energy perturbations typically involve simulations of 2 ns, over at least 21 intermediate states. We therefore feared that prohibitively long simulations would be required when relative binding free energies between different fragments are to be computed. For this reason, we used the more efficient A-EDS approach to study the preferences of the protein for six different fragments. In A-EDS, data for all compounds of interest are extracted from a single, longer, simulation, increasing the chances of capturing all relevant conformations throughout the simulation.

The A-EDS approach was used in two settings. After an optimization of the A-EDS parameters in solvent, a screening run was performed in the G12C mutant, directly identifying the three most promising compounds, in agreement with experimental measurements for the G12 V mutant. Further optimizing the A-EDS parameters in the protein as well, allowed for a quantification of the relative binding free energies, further confirming the experimental trends and the sampling times observed in the screening run.

## Conclusions

By using molecular dynamics simulations, the conformational flexibility and interaction potential of KRAS and its G12C mutant was studied. We rationalized the oncogenic stabilization of the active state of the G12C mutant in terms of molecular and thermodynamic observations. The considerable flexibility of the protein and differences in behavior between various relevant states (nucleotide or inhibitor binding) pose additional challenges for the evaluation of thermodynamic properties, such as the binding free energies. For this reason, we explored the possibilities of using alternative thermodynamic cycles and the use of a more efficient free-energy method.

The increased stability of the oncogenic active state of the KRAS could be traced to the enhanced interactions of Cys12 with the nucleotide GTP in the G12C mutant. A hydrogen bond between Cys12 and the γ-phosphate of GTP leads to stabilization of this state, which could be quantified by alchemically performing the mutation in the simulations. Furthermore, we used the A-EDS method to classify a set of six small fragments with respect to their binding affinity. The screening mode of A-EDS correctly identified the three most tightly binding fragments and further optimization of the A-EDS parameters in the bound state allowed us to quantify the relative binding free energies of the compounds.

Overall, our work shows how molecular simulations can contribute to the understanding of complex targets in molecular drug design. The A-EDS approach to screen for the affinity of a small set of binders is a promising approach that circumvents various issues in more elaborate free-energy methods. The best molecules may be selected from a set by performing a single simulation, without the need for a large amount of simulations or extensive optimizations of the parameters in the protein.

## Supplementary Material



## Data Availability

The simulations were performed using version 1.6.1 of the GROMOS simulation package https://www.gromos.net and version 2023.1 of the Gromacs simulation package https://www.gromacs.org. The input files required to run the MD simulations of the KRAS protein are openly available on Zenodo at 10.5281/zenodo.18183886 (accessed on 08 January 2026). These files include topologies, coordinates, and input files for all molecular simulations, as well as the scripts used for the analyses described in this work.

## Notes

Atom-position root-mean-square fluctuations of KRAS wildtype in the Apo and Holo states; occurence of hydrogen bonds between C12 in the G12C mutant of KRAS and the nucletodides; distance heatmaps between GDP or GTP and residues 10 to 37 of the wild-type Apo and Holo conformations; distance heatmaps between compound **12** and amino acids Asp57 to Ser106 of the G12C mutant in the presence of nucleotides; occurrence of hydrogen bonds between compound **12** and the wild-type; representative structures from the sI/II region found by MDpocket; average sampling times and relative binding free energies of the six fragments (PDF)

## ⊥

Department of Theoretical Biophysics, Max Planck Institute of Biophysics, Max-von-Laue-Straße 3, 60438 Frankfurt am Main, Germany
