# Supplementary material for: Unveiling the KRAS Relationship between Affinity and Dynamics: A Molecular Simulations Study
Source: J Chem Inf Model. 2026 Jul 16;66(14):8522–32. doi: 10.1021/acs.jcim.6c01239 (PMC13418165; doi:10.1021/acs.jcim.6c01239)
Supplement: Supplementary file 1 [file ci6c01239_si_001.pdf]

Supplementary Information

for

Unveiling the KRAS relationship between affinity and dynamics: A molecular simulations study

Nadine Grundschober, Viktorija Dujmovič, Chris Oostenbrink, Drazen Petrov, Zuzana Jandova, Andreas Bergner, Edgar Galicia-Andrés

BOKU University

Institute of Molecular Modeling and Simulation

Department of Natural Sciences and Sustainable Resources

Muthgasse 18, 1190 Vienna, Austria

Contents

Atom-position root-mean-square fluctuations of KRAS wildtype in the Apo and Holo states S2

Occurence of hydrogen bonds between C12 in the G12C mutant of KRAS and the nucletodides S3

Distance heatmaps between GDP or GTP and residues 10 to 37 of the wild-type Apo and Holo conformations S4

Distance heatmaps between compound 12 and amino acids Asp57 to Ser106 of the G12C mutant in the presence of nucleotides S5

Occurrence of hydrogen bonds between compound 12 and the wild-type S6

Representative structures from the sI/II region found by MDpocket S7

Average sampling times and relative binding free energies of the six fragments S8

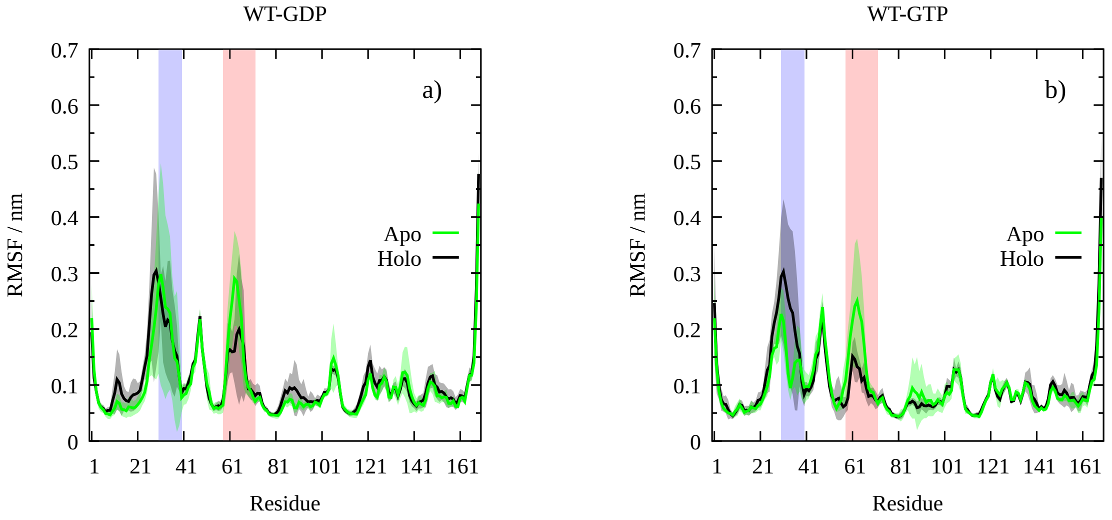

Figure S1. Atom-positional root-mean-square fluctuations of KRAS wildtype in the Apo (green line) and Holo (black line) states, in presence of nucleotide GDP (left panel; a) or GTP (right panel; b). The standard deviation over the five independent replicates is shown as shaded regions. Loop regions sI and sII are highlighted in blue and red, respectively.

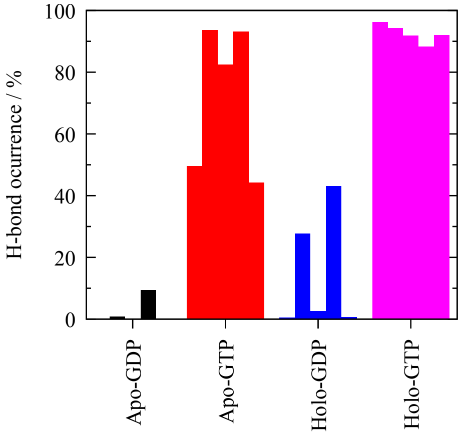

Figure S2. Occurrence of hydrogen bonds between Cys12 in the G12C mutant of KRAS and the nucleotide GDP or GTP in the Apo and Holo simulations. Individual bars represent the five replicate simulations for every combination of nucleotide and fragment binding.

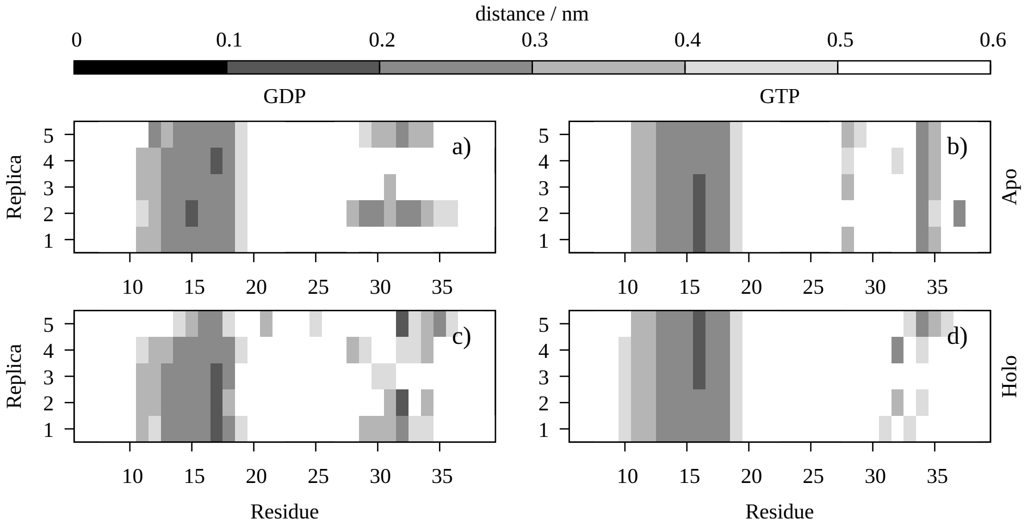

Figure S3. Distance heatmaps between nucleotides GDP (left panels) and GTP (right panels) and residue range 6 – 39 of the wild-type Apo (top panels) and Holo (bottom panels) conformations. The average of the shortest distance between the nucleotide and the indicated residues is highlighted for the five individual replicate simulations.

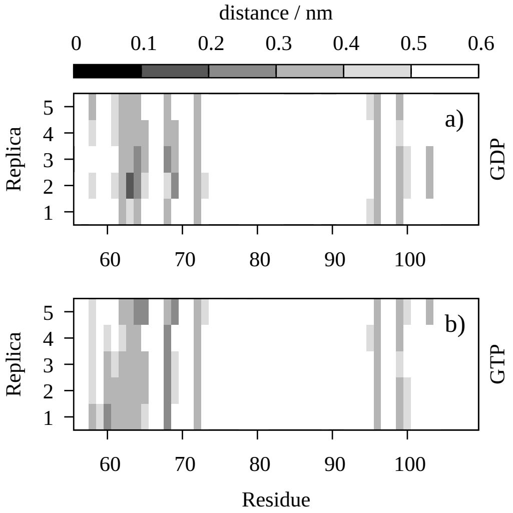

Figure S4. Distance heatmaps between compound **12** and amino acids in the residue range 57 – 106 of the G12C mutant in the presence of nucleotides GDP (top panel) or GTP (bottom panel).

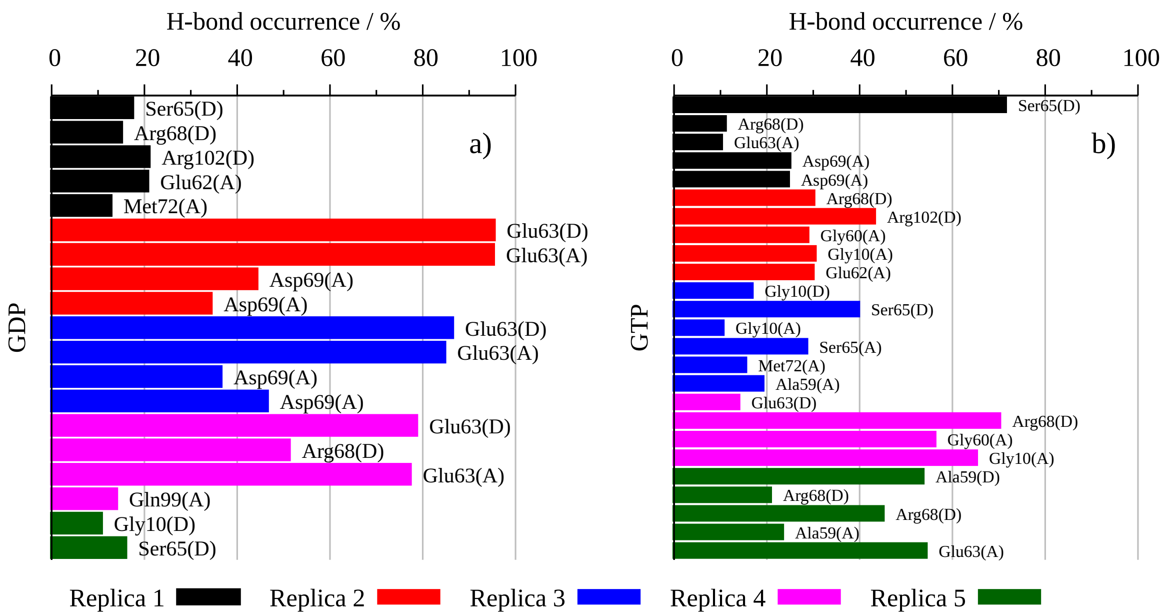

Figure S5. Occurrence of hydrogen bonds between compound **12** and the wild-type, in the presence of GDP (left panel) and GTP (right panel). The colors indicate individual replicate simulations.

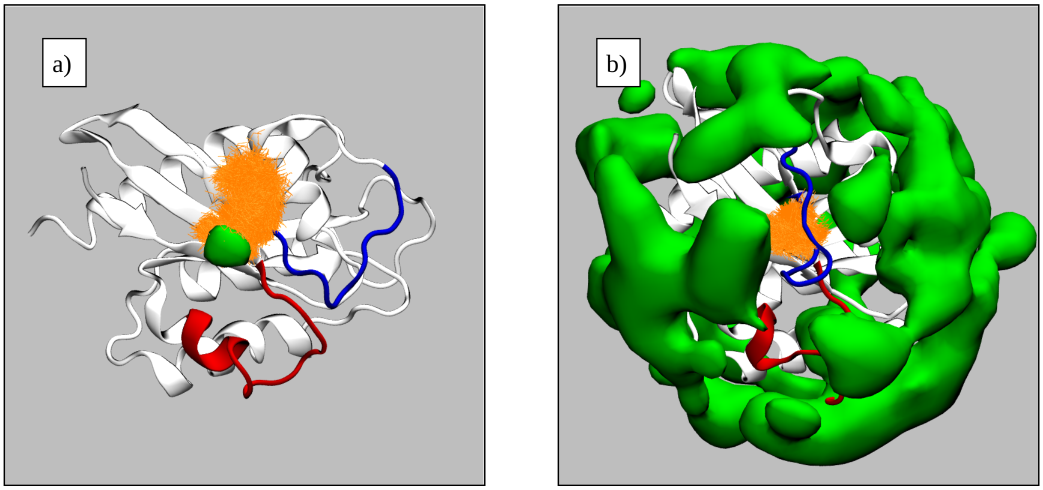

Figure S6. Representative structures from the sI/II region found by MDpocket with occurrence frequency in the simulation of: a) 50% (low volume values); and b) 20% (high volume values), corresponding to volume distributions of pocket sI/sII Holo (rightmost violin plot) in Figure 4a. Different conformations of Val39 are represented by orange lines for reference of the pocket.

Table S1. Average sampling times and relative binding free energies of the six fragments. The sampling times are obtained from the set of A-EDS parameters optimized in the solvent simulations, while for the relative binding free energies, the optimized set of parameters for the protein were used.

Compound	Sampling		$\Delta\Delta G_{bind}$
	Water [%]	Protein [%]	[kJ mol^{-1}]
**1**	16	1	0.5 ± 0.5
**3**	15	0	10.8 ± 2.5
**6**	15	0	4.4 ± 1.2
**11R**	18	24	-5.0 ± 1.5
**11S**	19	15	-4.2 ± 0.8
**12**	17	60	-8.7 ± 0.4
